# Improved Interlaminar Fracture Toughness and Electrical Conductivity of CFRPs with Non-Woven Carbon Tissue Interleaves Composed of Fibers with Different Lengths

**DOI:** 10.3390/polym12040803

**Published:** 2020-04-03

**Authors:** Feng Xu, Bo Yang, Lijie Feng, Dedong Huang, Min Xia

**Affiliations:** 1 School of Astronautics, Northwestern Polytechnical University, Xi’an 710072, China; huangdedong@nwpu.edu.cn; 2 Qingdao Research Institute, Northwestern Polytechnical University, Qingdao 266200, China; 3 Center for Advanced Materials Technology (CAMT), School of Aeronautics, Mechanical & Mechatronic Engineering J07, The University of Sydney, Sydney NSW 2006, Australia; 4 Shanghai Spaceflight Precision Machinery Institute, Shanghai 201600, China; 18916824056@163.com (B.Y.); 17717612936@163.com (L.F.); 5 Yangtze River Delta Research Institute, Northwestern Polytechnical University, Taicang 215400, China

**Keywords:** non-woven carbon tissues, interlaminar fracture, CFRPs laminates

## Abstract

Non-woven carbon tissue (NWCT) with different fiber lengths was prepared with a simple surfactant-assistant dispersion and filtration method and used as interleaving to enhance both delamination resistance and electrical conductivity of carbon fiber reinforced plastics (CFRPs) laminates. The toughing effect of NWCT on both Mode I and Mode II interlaminar fracture of CFRPs laminate is dependent on length of fibers, where the shorter carbon fibers (0.8 mm) perform better on Mode I interlaminar fracture toughness improvement whereas longer carbon fibers (4.3 mm) give more contribution to the Mode II interlaminar fracture toughness increase, comparing with the baseline composites, and the toughness increase was achieved without compromising of flexural mechanical properties. More interestingly, comparing with the baseline composites, the electrical conductivity of the interleaved composites exhibited a significant enhancement with in-plane and through-the-thickness direction, respectively. Microscopy analysis of the carbon tissue interleaving area in the laminate indicated that carbon fibers with shorter length can form into a 3D network with more fibers aligned along through-the-thickness direction compared with longer ones. The shorter fibers thus potentially provide more effective fiber bridges, pull-out and matrix deformation during the crack propagation and improve the electric conductivity significantly in through-the-thickness direction.

## 1. Introduction

Carbon fiber reinforced plastics (CFRPs) laminate have been widely used in weight-critical structures, such as aircraft, spacecraft, racing cars, etc., due to excellent mass-specific mechanical properties. Unfortunately, poor delamination toughness [1] in plies of composite laminate has become the important limiting factor in practical structure application. The extensive methods were obtained for improving the delamination toughness of CFRPs composites, including the techniques of toughening the matrix [2], Z-pins [3], stitching [4], and the surface modification of carbon fabrics [5,6] and interleaf [7]. Among these methods, the interleaving technique is one of the latest developed technologies, which keeps the simplicity in the manufacturing process. Current representative interleave methods can be classified into several strategies: (1) chemical synthetic interleaf film on fabric surface [8], (2) nanoparticles/thermoplastic or thermosetting film [9], (3) electrospinning nanofiber film [10], (4) nanoparticles film [11], (5) commercial non-woven tissue (NWT) [7], which all give the greatest contribution to interlaminar toughness improvement of the composite laminate. However, the first four methods still have some problems such as high dependence on some specialized equipment, low preparation efficiency and high cost, which are not applicable and realistic to practical application, instead, the commercial polymer interleaf materials have the advantages of mature production process, high production efficiency and low cost.

A simple and low-cost method of pioneer work to toughen laminate composites is to utilize short Kevlar fibers, initially proposed by Hu et al. [12,13,14] and followed by Park [15], who manually spread commercial Kevlar fibers with around 2–15mm in length in the mid-plane of laminates, where the significant fiber bridging was observed for the Mode II test [13], while Mode I exhibited lower fracture energies with less apparent fiber bridging [12]. However, the data for the laminates with short Kevlar fibers shows a wider scatter due to the uneven distribution of the Kevlar fibers, which probably comes from the manually spread method. Recently various commercial polymer films have been utilized as interleaving materials in CFRPs, such as chopped aramid fibers [16], polyimide and polypropylene fibers [17], polypropylene nonwoven fabric [18], silk fibers interleave [19] and resulted in varied success in the toughness improvement of the composites. However, the incorporation of commercial interleaf with insulating or soft fibers will lead to the reduction of the electrical conductivity of the laminates and possible decrease in-plane strength and modulus of the laminates [16] due to the high electrical insulation and weak mechanical properties of the polymer material in nature. Therefore, the commercial non-woven carbon tissue (NWCT) composed of rigid and conducting carbon fibers was utilized as interleaved reinforcement into CFRPs laminate to characterize their reinforcing effect for various mechanical properties such as Mode II [20] and I interlaminar fracture toughness [21], fatigue [22] and in-plane mechanical properties [23,24,25], here, it is noted that the NWCT with fiber length of 15 mm can significantly increase Mode II interlaminar fracture toughness of the laminated CFRPs composites by over 200% [20,26], without compromise the in-plane properties including stiffness, strength and fatigue life [22,23,24,25]. However, less improvement was found for the Mode I interlaminar fracture toughness using this interleave [21], even with the modification of the CNTs on the NWCT [26], where the fiber length here is long enough to be 15 mm [25], and thus most of fibers are more likely aligned along the in plane direction of the laminate, which is unfavorable for the Mode I toughness increase [21]. To overcome this problem, our previous work [27] has found that NWCT with shorter length fibers (approximately 0.8 mm in length) exhibited obvious enhancement for Mode I fracture toughness of CFRP laminate, indicating the fiber length is one of key parameters to determine the NWCT toughening effect in CFRP laminate. However, toughening mechanisms and discussion for Mode I interlaminar fractures are not sufficient, and how the NWCT with shorter fiber length prepared in our previous work affects Mode II interlaminar fracture, electrical conductivity and in-plane mechanical properties has not been reported so far.

The purpose of this paper is to prepare NWCTs with different fiber length and utilize them as the interleaves for the interlaminar fracture toughness and electrical conductivity improvement of laminated composites. It is expected that the structure parameters of carbon tissues in the interleaving layers in the laminate have great effect on the fracture toughness of the CFRPs laminate, since the aspect ratio, and alignment of the one dimensional fillers in the composites have much influence on the properties of the composites [28]. The effect of these parameters has never been revealed before, while the NWCT composed of only longer carbon fibers (15 mm) were studied previously [25]. The toughed mechanism for different length of non-woven carbon fiber tissue is discussed based on the microscopy observation. The chopped carbon fibers can be combined with pre-preging process to facilitate the end users to use the modified pre-pregs in a normal lay-up process. Thus, the fabrication technique of composites with chopped fibers reinforcement is validated in providing an applied prospect of the future research. 

## 2. Experimental Work

### 2.1. Raw Materials

Materials used in this study were woven carbon fibers (168058ITL supplied by Inter-Turbine Advanced logistics Pty Ltd., Sydney, Australia) for the CFRPs laminates composites and NWCF interleave preparation, the epoxy resin system including Araldite-F (diglycidyl ether of bisphenol A, DGEBA) and piperidine, supplied by Sigma-Aldrich (Sydney, Australia), and surfactant (cellulose, supplied by Sigma-Aldrich, Sydney, Australia).

### 2.2. NWCT Fabrication

**Figure 1:**
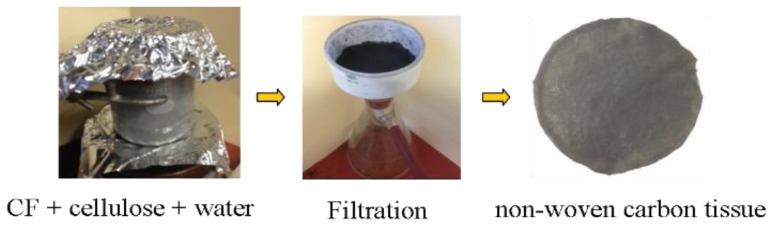
The procedure of preparing non-woven carbon tissue.

The woven carbon fibers were manually chopped into short carbon fibers (SCFs) with around 0.8mm in length and long carbon fibers (LCFs) with around 4.3 mm in length respectively. As shown in Figure 1, the aqueous dispersion containing chopped carbon fiber and surfactant was stirred for 60 min to prepare the uniform dispersion of chopped carbon fiber in the solution. After filtration of the dispersion and rinsed with distilled water to remove the residual surfactant, the final non-woven carbon tissue (NWCT) was obtained. The density of prepared SCF and LCF interleaves is 7.8 g/cm^2^, corresponding to the thickness of 150 μm in thickness respectively.

### 2.3. Laminates Preparation

**Figure 2:**
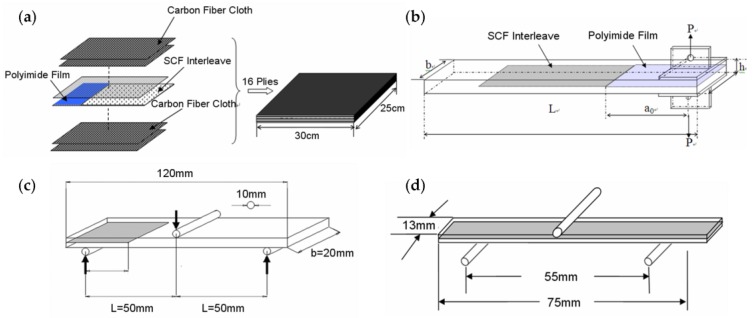
(**a**) Schematic for fabrication of non-woven carbon tissue (NWCT) interleaved laminates and the specimen geometry for (**b**) Double cantilever beam(DCB), (**c**) End Notched Flexure (ENF), (**d**) Flexture properties tests.

The CFRPs laminates composites were fabricated from 16 plies of plain-woven carbon fibers and neat epoxy by the hand lay-up method used before [29]. A 0.2 mm thick Kapton polyimide film and the prepared NWCT were inserted at the mid-plane of the laminates to serve as the pre-crack and interleave respectively to fabricate the sample for the interlaminar fracture toughness tests, illustrated as Figure 2. The laminates were wrapped with bleeders and release film within a vacuum bag, and first vacuumed in a chamber for 20 min followed by curing in a hot-press at 120 °C for 16 h. A pressure of 250 kPa was applied during curing to maintain a uniform laminate thickness and a constant fibre volume fraction, which were 3.1 mm and 60% ± 2%, respectively. Double cantilever beam(DCB)and End Notched Flexure (ENF) specimens were finally cut from the square panels by a wet-jet diamond saw. In addition, two-ply laminates interleaved with NWCT, were also prepared with the same method above for the electric conductivity measurement.

### 2.4. Experimental Procedure

All the mechanical properties tests were performed on an Instron 5567 machine (ITW, Boston, MA, USA). Mode I DCB interlaminar fracture toughness was conducted according to ASTM Standard D5528 [30] to further study NWCT toughening mechanism. As shown in Figure 2b, the initial crack length is 40 mm. The crack mouth opening displacement rate was 2 mm/min. The load-displacement curves were recorded and crack growth was monitored with a travelling microscope. Delamination toughness, *G*_IC_, was determined by the Modified Beam Theory (MBT) Method, which was recommended by the Standard [30], that is:

(1) $$G_{IC}=\frac{3P\delta }{2b(a+\left|\Delta \right|)}$$

where *P* is applied load, *δ* is displacement of the load-point and |Δ| is modification of measured crack length.

Standard 3-point ENF tests were performed in an Instron 5567 machine according to the Protocaol for Interlaminar fracture Testing No.2 (1992) [31]. As Figure 2c shown, the initial crack length a is 25 mm and *a*/*L* = 0.5. The crack mouth opening displacement rate was 2 mm/min. At least 4 samples were tested for each matrix system and their load-displacement curves were recorded. The interlaminar toughness *G*_IIC_, was calculated according to the protocol [31] by:

(2) $$G_{IIC}=\frac{9a^{2}\delta P}{2b(2L^{3}+3a^{3})}\cdot \frac{1-0.6099\times {\left(\delta /L\right)}^{2}}{1+0.3766\times {\left(\delta /L\right)}^{2}}$$

where *δ* and *P* are displacement and maximum force recorded at the load-point at fracture.

The flexural properties of the CFRP laminate composites were determined from the three-point bending test according to ASTM D790 [32]. Rectangular specimens of 75 mm long × 13 mm wide × 3.3 mm thick were loaded with a span of 55 mm at a crosshead speed of 1.4 mm/min, as shown in Figure 2d. Five specimens were tested for each set of conditions. The conductivity of the interleaved laminates in both in-plane and through-the-thickness direction were measure with a CHI electrochemical workstation. To improve the electrical contact, silver paste was applied on certain sides of the samples.

### 2.5. Microstructure Analysis

The crack propagation path and the orientation of chopped carbon fiber in the matrix in the mid-layer of laminate were observed by the optical microscopy (OM, Leica Microsystems Inc, Buffalo Grove, IL, USA). The fracture surface of the samples was coated with a thin gold layer and their morphologies were studied by SEM (Zeiss ULTRA Plus SEM, Zeiss, Oberkochen, Germany) at an accelerated voltage of 2kV.

## 3. Results and Discussion

### 3.1. The Fabrication of NWCT Made of the Chopped CFs with Different Length

**Figure 3:**
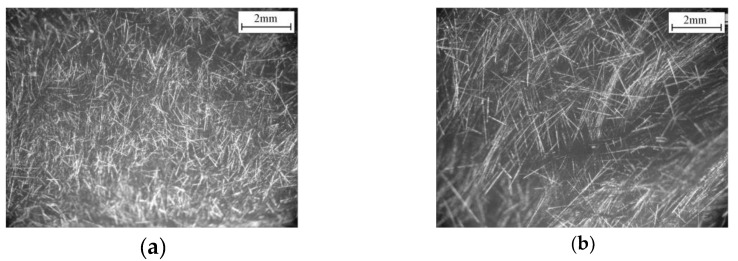
The optical images of NWCT made of chopped carbon fibers (**a**) short carbon fibers (SCFs), (**b**) long carbon fibers (LCFs).

Either the chopped SCFs or LCFs can be well dispersed in the solution in the presence of the surfactant. After filtration, NWCTs composed of fibers with different length was obtained, as shown in Figure 3. It can be found that the chopped carbon fibers are uniformly and randomly distributed in the NWCT and no fiber bundles/aggregation was observed, indicating that the original fiber bundles in the plain-woven fabrics were exfoliated by the present nonionic surfactant in the solution and single fibers were well separated from each other. It is believed that the surfactant plays an important role as stabilizer through the non-covalent polymer wrapping to prevent the formation of bundles or aggregation, just like their role in the preparation of the stable carbon nanotube dispersions. The mean length of LCFs and SCFs are 4.2 ± 0.5 mm and 0.8 ± 0.2mm respectively according to the statistics of 100 carbon fibers.

### 3.2. The Distribution of Chopped Carbon Fibers in the Mid-Layer of Laminate

**Figure 4:**
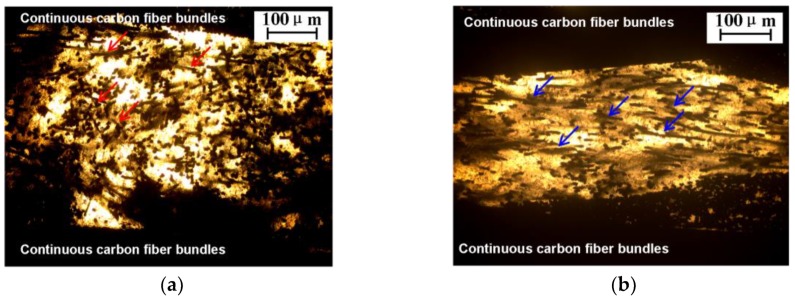
The optical images of the mid-layer of laminates interleaved with NWCTs made of (**a**) SCFs; (**b**) LCFs.

The NWCTs made of different fiber length were directly used as interleaves in the CFRPs laminates. Figure 4 shows the distribution of chopped carbon fibers in the mid-layer of laminate. For the NWCT interleaves made of SCFs, as red arrow indicated in Figure 4a, it can be seen that most SCFs are randomly dispersed and formed as the three-dimensional interwoven network structure, which would help to prevent the crack propagation efficiently. In addition, the SCFs enlarge the distance of adjacent layer of laminate, which help to increase the plastic zone of crack tip. In comparison, most LCFs (blue arrow indicated) are aligned along the in-plane direction due to their larger length.

### 3.3. Mode-I Interlaminar Fracture Toughness of NWCT-Composites

**Figure 5:**
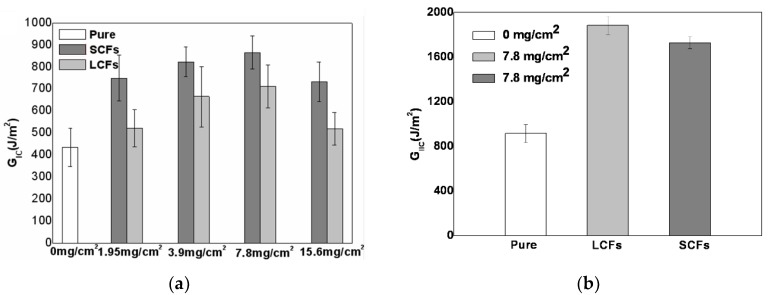
The dependence of (**a**) Mode I and (**b**) Mode II interlaminar fracture toughness of the interleaved CFRPs laminates on the density and fiber length of the NWCT.

The influence of NWCT interleaves with different fiber length and density on the Mode I interlaminar fracture toughness improvement was studied systematically in our previous study [27], as shown in Figure 5a, the fracture toughness of the NWCT interleaved laminates was increased gradually with the increasing of the density of NWCTs from 1.95 to 7.8 mg/cm^2^. For the laminates interleaved with the NWCT made of SCFs, the maximum *G*_IC_ was 865 J/cm^2^, which is a remarkable 99% increase compared to the baseline composite. However, further increasing the NWCT interleaves’ density to 15.6 mg/cm^2^ leads to the decrease of *G*_IC_, although the value still bigger than that of the baseline composite. Similar trends of the toughness dependence on the density also happened on the laminates interleaved with NWCTs made of LCFs, where the longer carbon fibers diminish their positive effect on the Mode I fracture toughness enhancement. These results imply that the NWCTs made of SCFs can improve Mode I fracture toughness more effectively than those made of LCFs.

To put the Mode I toughness results obtained in this work in perspective of similar studies by other researchers, relevant toughness data was compiled from the literature in Table 1 for easy comparison, in which the Mode I interlaminar fracture toughness for CFRP composites was modified by various carbon materials such as NWCT tissue with longer fiber length (15 mm) [20,25], CNTs grafted NWCT [26], carbon nanofibers [11] and brushing and abrading carbon fibers [29]. Since all materials have different CFRPs types and fiber content, it is more sensible to compare the percent increase of the plateau toughness relative to that of neat CFRPs as the baseline. Hence, from Table 1, it is clear that the enhancement of *G*_IC_ value obtained in our NWCT modified CF/E composite is obviously among the best results, indicating that with proper length a density, the NWCT with commercial micro-fibers are capable to significantly improve Mode I interlaminar fracture toughness of CFRPs laminate for structural applications, which is even much more effective than some trending CNTs bucky paper [33], carbon nanofibers [11] and CNTs grafted NWCT tissues [26]. Although types of NWCT tissue prepared by Lee [22] and our group are composed of carbon fibers, they display different morphology in CF/E composite. The longer carbon fiber (15 mm) of NWCT is mainly aligned in the in-plane direction of the laminates, the similar morphology of can be found in Figure 4b, where the fiber length is 4.3 mm. By contrast, the shorter carbon fiber (0.8 mm) of NWCT in this work within the interlayer are more easily to be distributed randomly in 3D directions, evidenced by Figure 4a, which demonstrates efficient crack bridging for toughness improvement. 

### 3.4. Mode-II Interlaminar Fracture Toughness of NWCT-Composites

As the optimum density of NWCT interleaves for the Mode I fracture toughness improvement is 7.8 mg/cm^2^, the NWCTs with the same density was prepared for the study on their interleaving effect on Mode II fracture toughness of the laminate. Figure 5b compares the Mode II fracture toughness *G*_IIC_ of pure CFRPs and those interleaved with NWCTs made of fibers with different length. Notably, the incorporation of NWCTs with both fiber lengths can significantly increase the Mode II fracture toughness of CFRPs laminates, which is increased by 105% for NWCT interleaves made of LCFs and 88% for those made of SCFs respectively. In contrast to the case of the Mode I fracture toughness, as summarized in Table 2, interleaves composed of carbon fibers with longer length such as NWCT tissue (15 mm), CNTs grated NWCT (15 mm) and carbon nanofibers exhibit better effect on the Mode II fracture toughness improvement than our NWCT interleave with shorter fiber length (0.8 and 4.3 mm), indicating that the NWCTs made of longer fibers can improve Mode II fracture toughness more effectively than those made of shorter fibers. 

### 3.5. Flexural Properties of Short Chopped Carbon Fiber Reinforced Laminate

**Figure 6:**
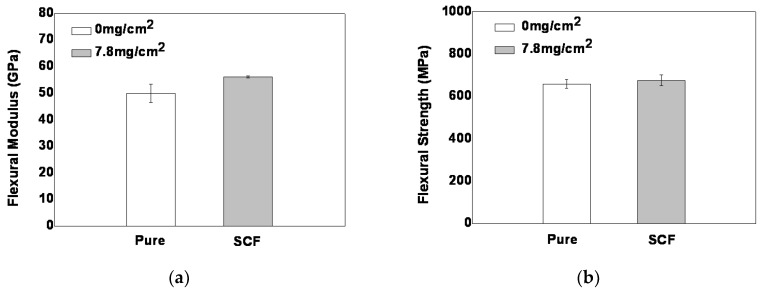
(**a**)Flexural modulus and (**b**) flexural strength of NWCT reinforced laminate.

Since the out-plane properties such as Mode I and Mode II interlaminar fracture toughness were significantly improved by the NWCT with density of 7.8 mg/cm^2^ and length of 0.8 mm, the representative flexural mechanical properties of CFRP laminate interleaved by this NWCT is obtained as shown in Figure 6, where the flexural strength and modulus of laminate were increased by 2.5% and 12% over the baseline composite respectively. The improvement of the flexural strength of laminate is owing to the stress transfer between the matrix and SCFs in the mid-layer.

### 3.6. The Conductivity of the NWCT Modified Laminates

**Figure 7:**
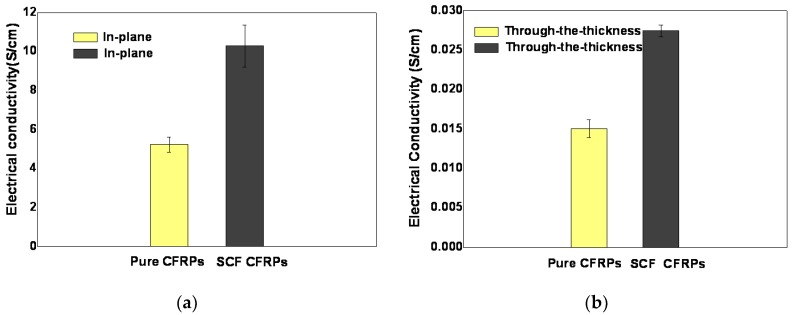
The electrical conductivity of the laminates in different direction (**a**) In-plane; (**b**) Through-the-thickness.

Figure 7 shows the electrical conductivities of the laminates interleaved with NWCTs made of SCFs. The carbon fabric plies used in the composites is 0°/90° plain woven fabrics, which make the electrical conductivity of the composite quite different in the direction of in-plane and through-the-thickness of the laminates. The in-plane electrical conductivities were two magnitude orders higher than those in through-the-thickness direction, as shown in Figure 7a,b. Such huge difference mainly results from the laminated structure of the CFRPs composite. While continuous carbon fibers of the carbon fabrics are aligned along in-plane direction and directly build up the conductive network in this direction, the highly resistive epoxy resin-rich area is always located between carbon fabric layers, resulting in the reduced electrical conductivity in the through-the-thickness direction. Compared with the baseline CFRPs laminates, after the incorporation of NWCTs with density of 7.8 mg/cm^2^, the in-plane electrical conductivity was increased by over 96% (Figure 7a), while the through-the-thickness electrical conductivity was increased by over 82% (Figure 7b). which are higher than that of CRRP interleaved by other carbon interleave materials such as black@PPNWF [18] and CNTs-doped polyamide [9] in the literature, as shown in Table 3, indicating excellent electrical conductivity of the NWCT with commercial short carbon fiber as for the conductive interleave in CFRPs laminate. Obviously, the increase in electrical conductivity is due to the connected conductive network of SCFs with high density (7.8 mg/cm^2^) in the matrix between fabrics plies, evidenced by Figure 4a.

### 3.7. The Crack Propagation Path in the Mid-Layer

**Figure 8:**
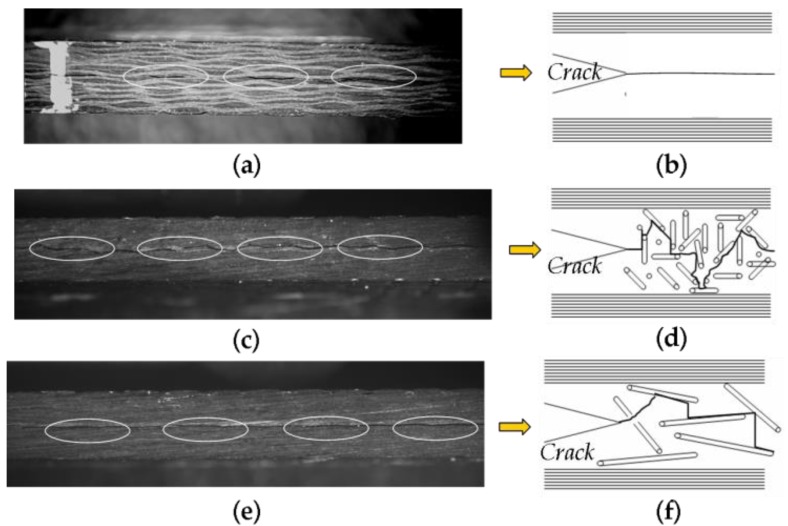
The crack propagation path in the mid-layer of laminates during Mode I DCB tests: (**a,b**) pure; (**c,d**) SCFs (7.8 mg/cm^2^) and (**e,f**) LCFs (7.8 mg/cm^2^).

Figure 8 shows the crack propagation path in the mid-layer of laminates after DCB tests. As shown in Figure 8c, laminate interleaved with NWCTs made of SCFs shows twisty crack propagation path as shown in white circles compared with pure CFRPs laminate as shown in Figure 8a,b. This crack propagation process can absorb more fracture energy and leads to larger fracture area, and finally results in the high fracture toughness improvement. Figure 8d further shows that the twisty crack propagation path may come from the three-dimensional interwoven network structure of SCFs in the matrix of laminate. Figure 8e gives the crack propagation path of laminate interleaved with NWCTs made of LCFs. 

It can be seen that the crack propagates though the interleave tissue and the crack deflection can also been observed in the mid-layer as shown in solid circles. However, the extent of the crack deflection for the interleaves made of LCFs was less than that of NWCTs made of SCFs which results in the reduced toughness improvement. This comparison indicates the NWCTs made of SCFs afford higher efficient crack bridge effect than that made of LCF for the crack propagation in Mode I fracture toughness tests. Figure 8f further suggests moderate crack deflection can attribute to LCFs alignment in-plane direction.

### 3.8. Toughening Mechanism Discussion

**Figure 9:**
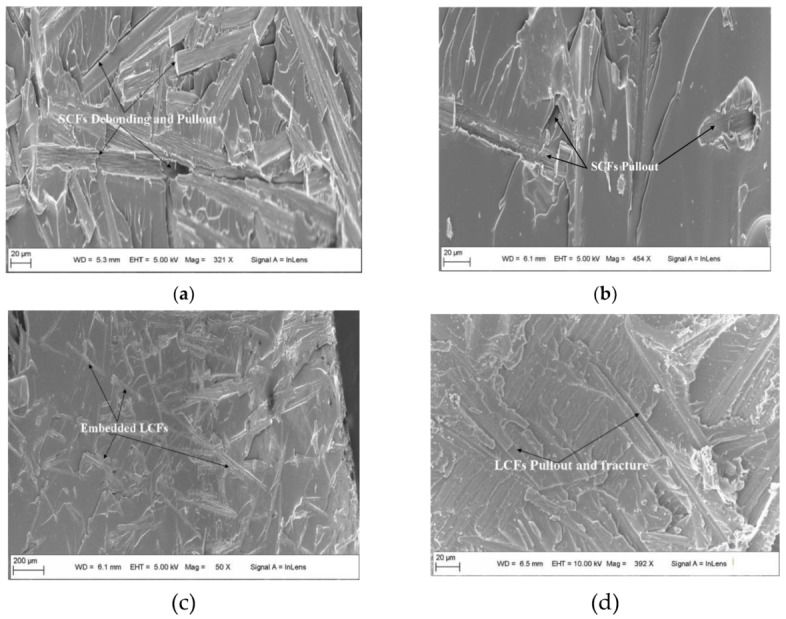
The fracture surface morphology of the laminates interleaved with NWCT made of (**a**,**b**) SCFs for Mode I, (**c**) LCFs for Mode I and (**d**) LCFs for Mode II.

The fracture morphology of NWCTs interleaved laminates was given in Figure 9. As shown in Figure 9a, the SCFs was randomly distributed on the fracture surface of laminates after the delamination, these SCFs were debonded from the matrix as shown in Figure 9a (black arrows), which make the surrounding matrix large deformation, with evidence of different fracture surface steps of epoxy resin and sub-microcrack on the fractured epoxy resin. The rougher fracture surface with different fracture surface steps was attributed to the crack deflection around short carbon fibers, as these processes significantly absorb fracture energy. The fracture surface morphology with another feature was shown in Figure 9b, the sword and sheath can be clearly seen as the arrow indicated, which indicated the SCF pullout from the matrix. The frictional pullout process from the matrix can greatly absorb much more energy resulting in fracture toughness improvement. As for LCFs, different fracture morphology can be seen in Figure 9c, most of LCFs were embedded in the matrix and aligned along the in-plane-direction. This means only a small amount of LCFs was pullout and debonded from the matrix. Actually, compared with SCFs, LCFs embedded in the matrix were hard pullout or debonded due to the large shear force between the fiber and the matrix, as these will cause LCFs’ fracture in the matrix, which result in smooth fracture surfaces with less matrix deformation around the fibers, leading finally to the reduced toughness improvement.

An overview of the Mode II delamination surface of a specimen interleaved with NWCTs is shown in Figure 9d. The chopped carbon fibers are those without any fixed pattern on the left side of Figure 9d, where many of them have been torn away from their original positions, and they were pulled out due to shear traction stress during delamination with evidence of long sheath left on the fracture surface. It can also be observed that the chopped carbon fibers show the crack bridging effect with evidence of fractured carbon fibers. The pullout or fracture behavior of chopped carbon fibers can cause the matrix deformation finally leading to rougher fracture surface and improved dissipated fracture energy.

## 4. Conclusions

The commercial non-woven carbon tissues (NWCT) with different fiber length were prepared though a simple surfactant-assistant dispersion and filtration method and used as interleaves to comprehensively enhance interlaminar fracture toughness and electrical conductivity of CFRP composites laminates without compromising of its flexural mechanical properties. Corresponding toughening and conductive mechanisms of NWCT with different fiber lengths were finally revealed by microscopy observation and analysis with some important findings, as follows:

(1) The NWCT made of shorter carbon fibers (0.8 mm) perform better on Mode I interlaminar fracture toughness than those made of longer carbon fiber (4.3 mm), achieving a significant Mode I toughness increase. However, longer carbon fiber (4.3 mm) give more contribution to the Mode II interlaminar fracture toughness.
(2) The electrical conductivity of composites interleaved by NWCT composed of shorter carbon fibers (0.8 mm) with high density of 7.8 mg/cm^2^ achieve an enhancement of 96% in the in-plane and 82% in the through-the-thickness direction, respectively, exhibiting the significant electrical conductivity improvement though the establishment of connected conductive network of SCFs in the mid-layer of laminate.
(3) Microscopy analysis of the NWCT interleaving area in the laminate zone indicate that the shorter chopped carbon fibers are more easily to form into a 3D network with more fibers aligned along through-the-thickness direction compared with those longer ones, causing more effective fiber bridges induced by fiber pullout and matrix deformation during the crack propagation.

## Figures and Tables

**Table 1:** Improvements in Mode I fracture toughness of CFRP composites with carbon interleaves.

Interleave	Test Method	Fracture Toughness Improvement ^1^	Ref.
Interleave with NWCT tissues (15 mm)	DCB	28%	[20]
Interleave with NWCT tissues (0.8mm)	DCB	99%	This Work
Interleave with CNTs grafted NWCT tissues	DCB	35%	[26]
Brushing and abrading carbon fibers	DCB	83%	[29]
Interleave with CNTs bucky paper	DCB	51%	[33]
Interleave with Carbon nanofibers	DCB	50%	[11]

^1^ % Improvement is relative to the same CFRPs without interleave.

**Table 2:** Improvements in Mode II fracture toughness of CFRP composites with carbon interleaves.

Interleave	Test Method	Fracture Toughness Improvement	Ref.
Interleave with NWCT tissues (15 mm)	ENF	26–260%	[21]
Interleave with NWCT tissues (4.3 mm)	ENF	105%	This Work
Interleave with CNTs grafted NWCT tissues	ENF	246%	[26]
Interleave with Carbon nanofibers	ENF	200–300%	[11]

**Table 3:** Increase in electrical conductivity of CFRP composites with various conductive intealeave.

Conductivity Interleave	Increase in In-plane conductivity	Increase in Out-of-plane conductivity	Ref.
NWCT tissues (0.8mm)	96%	82%	This Work
carbon black@PPNWF	/	unchanged	[18]
CNTs-doped polyamide	16%	<5%	[9]
